# Microwave-stimulated superconductivity due to presence of vortices

**DOI:** 10.1038/srep09187

**Published:** 2015-03-17

**Authors:** Antonio Lara, Farkhad G. Aliev, Alejandro V. Silhanek, Victor V. Moshchalkov

**Affiliations:** 1 Dpto. Física Materia Condensada C03, Instituto Nicolas Cabrera (INC), Condensed Matter Physics Institute (IFIMAC), Universidad Autoenoma de Madrid, 28049, Madrid, Spain; 2 INPAC- Katholieke Universiteit Leuven, Celestijnenlaan 200D, B3001, Leuven, Belgium and Departement de Physique, Université de Liège, B-4000 Sart Tilman, Belgium; 3 INPAC- Katholieke Universiteit Leuven, Celestijnenlaan 200D, B3001, Leuven, Belgium

## Abstract

The response of superconducting devices to electromagnetic radiation is a core concept implemented in diverse applications, ranging from the currently used voltage standard to single photon detectors in astronomy. Suprisingly, a sufficiently high power subgap radiation may stimulate superconductivity itself. The possibility of stimulating type II superconductors, in which the radiation may interact also with vortex cores, remains however unclear. Here we report on superconductivity enhanced by GHz radiation in type II superconducting Pb films in the presence of vortices. The stimulation effect is more clearly observed in the upper critical field and less pronounced in the critical temperature. The magnetic field dependence of the vortex related microwave losses in a film with periodic pinning reveals a reduced dissipation of mobile vortices in the stimulated regime due to a reduction of the core size. Results of numerical simulations support the validy of this conclusion. Our findings may have intriguing connections with holographic superconductors in which the possibility of stimulation is under current debate.

Microwave (*mw*) irradiation has been used to control the quantum properties of different systems, from supercurrents in superconductors to mechanical oscillators^1^,^2^,^3^,^4^. Using nonequilibrium pumping for cooling is currently a hot topic^5^,^6^. In 1966 microwave stimulated superconductivity (MSSC) was discovered^7^ in superconducting bridges and later confirmed for different type I superconducting systems such as films^8^,^9^, tunnel junctions^10^ or cylinders^11^,^12^. This counterintuitive effect was explained by Eliashberg^13^ as a consequence of an irradiation-induced redistribution of quasiparticles away from the gap edge. Very recently MSSC has been observed in transient regimes (on *ps* time scales) in NbN films^14^ and was demonstrated to improve the quality factor of superconducting *mw* resonators^15^.

In type II superconductors with magnetic field penetrating in form of quantized flux (vortices)^16^, the reduced inelastic relaxation time could suppress or modify some signatures of MSSC. A renewed interest in the type II SCs is related with the proposal of holographic superconductors (HS)^17^, mapping solutions of astrophysics problems to scalar condensates. Just like as type II SCs in solid state, HSs can exhibit vortex configurations^18^ and can be phenomenologically described by the time dependent Ginzburg-Landau equation (TDGL)^19^ in the proximity of the critical temperature. The possibility of stimulated SC in the HSs is under current debate^20^,^21^. Clearly, the experimental verification of stimulated superconductivity in type II SCs in the vortex state could therefore have important implications both inside and outside condensed matter physics community, paving the way for further progress in modelling physics of black holes and gravity through HSs.

A periodic *mw* pump of sufficient amplitude induces the motion of vortices that results in dissipation^22^,^23^,^24^,^25^. Though dynamics of vortices was extensively addressed^26^, the possibility of MSSC in the vortex state is not fully understood. One can speculate that the energy balance in microwave-driven vortices should depend on a competition between friction-induced heating of quasiparticles in the vortex cores^27^,^28^,^29^ and energy pumping outside the core at large vortex velocities^30^. However, the full picture of nonlinear electromagnetic response of the vortex matter in the proximity to the critical temperature remains unsettled.

Our paper reports on broadband nonlinear response to *mw* radiation in the GHz range in type II superconducting Pb films. We observe experimentally MSSC in an enhancement of the critical temperature, while much larger effects are seen in the second critical field. In order to investigate stimulation under varying pinning strength (i.e. vortex motion amplitude), we have carried detailed studies of MSSC in the Pb films with periodic pinning centers (Pb-PCC) and with applied DC field inclined about 4° off the film plane. Such configuration has been chosen because the most pronounced effect has been observed with it. Besides, the small perpendicular field component *H*_⊥_ creates vortices, permitting us, by properly chosing the magnetic field intensity, to achieve the situations where the number of vortices is an integer multiple of the number of pinning centers (matching conditions). In that case, the vortex lattice rearranges itself in a specially stable manner, and vortex motion becomes restricted. Therefore, the commensurability between the vortex lattice and the periodic pinning centers facilitates the investigation of the complex vortex dynamics under periodically varying pinning conditions. The experiments unambiguously reveal a reduction of the dissipation of microwave driven mobile superconducting vortices at moderate frequencies and powers. Supported by TDGL simulations, we relate this unexpected behaviour to the reduction of the vortex core size at large vortex velocities, predicted by Larkin and Ovchinnikov (LO)^30^ and seen indirectly when the flux is driven with a DC current^31^.

## Results

### Stimulation of critical temperature and upper critical field

**Figure 1:**
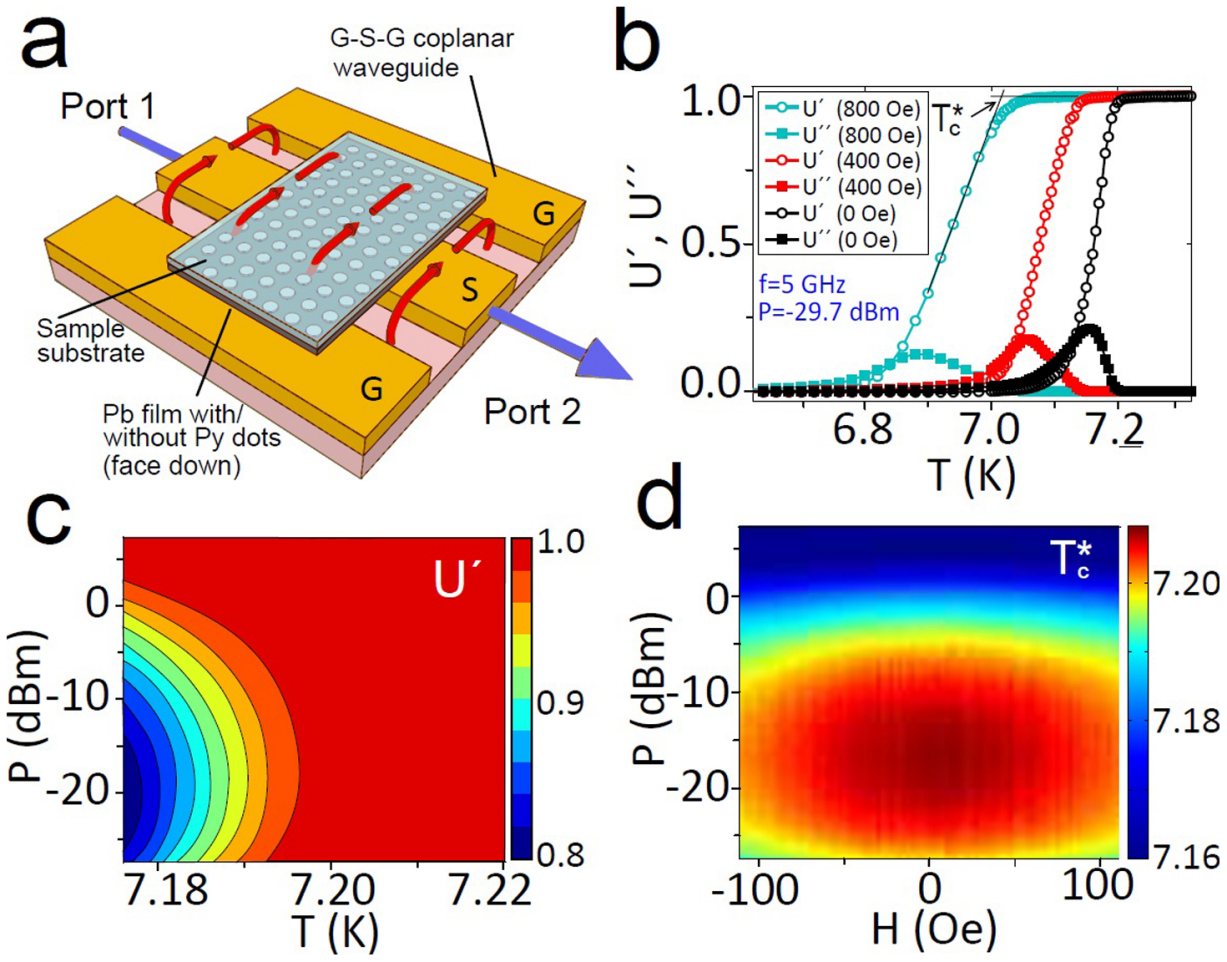
Sketch of the sample and effect of stimulation on critical temperature. (a) Sketch of the sample placed face down on the ground-signal-ground (G-S-G) coplanar waveguide (CPW). Red arrows represent the magnetic field generated by the CPW. (b) Measurements of *U*′ and *U*″ at different fields. (c) Contour plot of *U*′ versus microwave power and temperature, measured at *f* = 6 GHz. d) 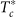 in the field range from *H* = −120 Oe to *H* = 120 Oe. All data correspond to the Pb-PPC sample.

A description of the samples and measurement details are provided in the Methods section and in the Supplementary material. Figure 1b shows the real and imaginary parts of the microwave permeability parameter *U*, defined in the Methods section, measured in Pb-PPC at small *mw* powers. Measurements are done in a temperature (*T*) sweep for different magnetic fields, at a fixed frequency (*f*) and are in accordance with the Coffey-Clem model^33^. The dependence of the response on the *mw* power (*P*) for the same Pb-PPC sample is seen in the contour plot for *U*′ in the plane *P* − *T* for magnetic field *H* = 0 and *f* = 6 GHz, (Fig. 1c). To characterize the shift of the transition as a function of *P*, we introduce an effective critical temperature, 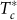, (Supplementary Fig. S3), determined with an error around 1 mK. Figure 1d presents a 3D plot of 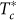 in the coordinates *P* − *H* in the Pb-PPC at *f* = 6 GHz. One observes a non-monotonic dependence of 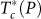, increasing at small *P* and decreasing at large *P*. Though the 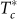 does not have the meaning of critical temperature of the superconducting transition, the observed increase of 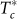 can be interpreted as an indication of MSSC.

**Figure 2:**
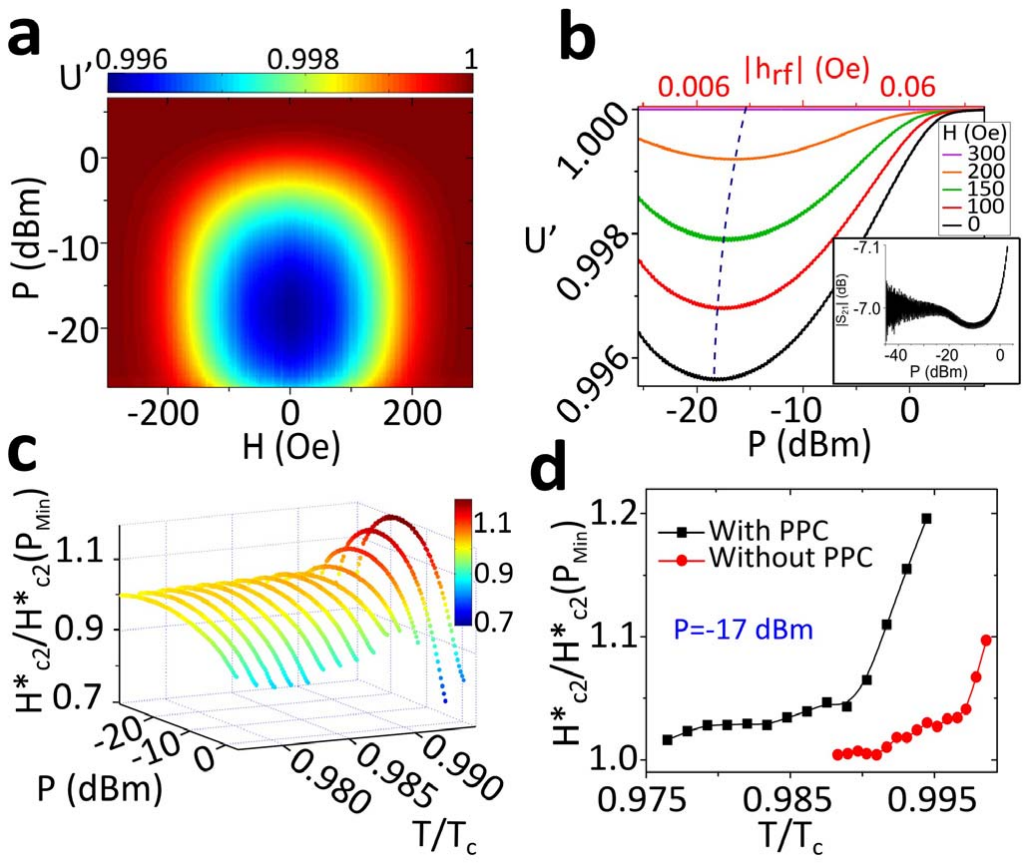
Effect of stimulation on the upper critical field. (a) Real part of the microwave permeability at *T* = 7.19 *K*, as a function of applied field and microwave power. (b) Cross sections of panel a) at fixed fields. The dashed line indicates optimum power. The inset shows a |*S*_21_| trace in a broader power range, at *H* = 0 Oe, *f* = 4 GHz and *T*/*T_c_* = 0.991. (c) 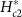 for different *T* and *P* for the Pb-PPC sample. Values are normalized at each *T* by the values of 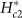 at the minimum power. (d) Comparison of normalized 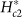 for each sample.

To investigate the nonlinear response in the vortex state as a function of pinning strength, we have carried out measurements of *U*′ and *U*″ as functions of *H*, *P*, *f* and *T* in Pb-PPC. Figure 2a shows a 3D plot of *U*′ for the Pb-PCC sample at *T* = 7.19 K in the coordinates *H* − *P*. Dark red tones correspond to the normal state. As *H* is lowered, the samples become superconducting and the magnetic permeability changes in agreement with expectations^33^. Figure 2b shows a typical set of cross-sections of *U*′ at fixed magnetic fields. While *mw* power above 5 dBm destroys superconductivity, for intermediate values the superconducting response is the most intense. We will refer to the applied *P* that yields the strongest superconducting response as “optimum power” (*P*_O_). The dashed line indicates how *P*_O_ changes with *H*. The response (inset of Fig. 2b) shows directly a transition between linear to nonlinear vortex response regimes. At lowest powers it attenuates and is noisier, since the relative noise is larger compared to the weaker signal received in port 2 of the network analyzer.

**Figure 3:**
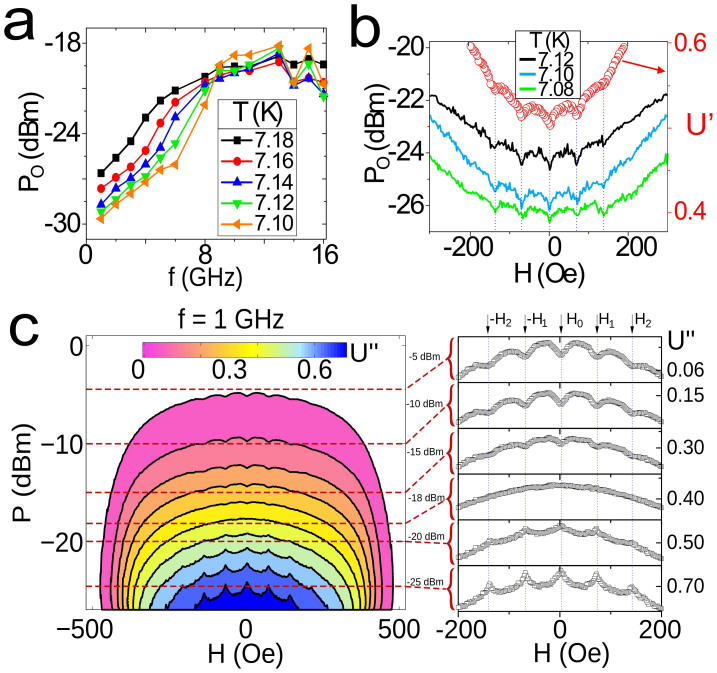
Nonlinear response of vortex losses at matching conditions. (a) *P*_O_ versus *f*. (b) Lines represent optimum power (*P*_O_) as a function of applied field at *f* = 6 GHz. Red circles (right axis) represent *U*′. Minima in *P*_O_ appear at matching fields. *U*″ in a power sweep is shown for *f* = 1 GHz in panel (c). The right part are cross sections at fixed powers. All panels correspond to the Pb-PPC sample.

Similarly to 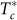, we introduce an effective critical magnetic field, 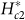. Figure 2c shows a plot of 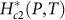 in the Pb-PPC sample (see Suplementary material for the method of finding 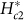, and the results for the plain film). For each temperature, 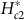 has been normalized by its value at the lowest *P*, so relative values of 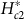 can be compared. As *T* → *T_c_*, the relative increase of 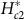 under *mw* at *P* = *P*_O_ becomes larger. Figure 2d compares the normalized 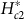 as a function of reduced temperature in both types of samples (with and without pinning centers), showing that MSSC effects in 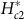 are stronger in the Pb-PPC sample. Figure 3a shows that *P* = *P*_O_ increases with *mw* frequency with a maximum value that saturates around 15 GHz. These frequencies are well below those corresponding to the superconducting gap.

### Reduced dissipation of microwave driven mobile vortex

The changes in *P*_O_ indicate that the “*cooling*” effectiveness of *mw* radiation depends on the pinning strength through the applied field which changes the number of vortices per pinning center and correspondingly their mobility. In Fig. 3b this fact is exposed for *f* = 6 GHz, at different values of *T*. Solid lines show *P*_O_ vs. magnetic field with matching conditions indicated by vertical dotted lines. Red circles represent a typical measurement of *U*′(*f*, *P*, *H*) at fixed values of *f* and *P*, in which the same matching anomalies appear in the *mw* permeability. The lowest value of *P*_O_ for every temperature is found always at zero field, and decreases locally in matching conditions. This hints the relevance of the vortex mobility for the value of *P*_O_: the enhanced vortex mobility out of matching conditions provides relatively larger (respect to matching) *P*_O_ values and correspondingly larger “cooling” efficiency.

This counterintuitive result has been corroborated through a set of independent experiments investigating the *H* and *P* dependencies of *U*″. At low *f*, when MSSC is not yet pronounced, the dissipation (*U*″) at matching conditions shows (as expected) dips in a broad range of *P* (Supplementary Fig. 7). However, for higher *f* the dips of losses at matching conditions convert into peaks. The same effect can be observed as a function of power, in Fig. 3c. In other words, *vortices moving with higher average velocities out of matching conditions manage to dissipate less than pinned in matching conditions*. These observations indicate a qualitative change in the microwave response of superconducting vortices at high *mw* frequencies.

## Discussion

### Mechanism of nonlinear vortex response and modelling

Mechanisms of nonlinear response of vortices to microwave radiation are far from being fully understood. LO theory^30^ predicts a nonlinear response at sufficiently large electric fields, that induces a high speed DC motion of the vortices. If their speed exceeds some critical value, *v_c_*, much lower than the critical velocity for breaking Cooper pairs, the current decreases with increasing voltage. This is a consequence of an electronic instability of the non-equilibrium distribution of quasiparticles at high velocities, leading to a reduction of the vortex core size. A further increase of vortex velocity leads to an abrupt switching into a state with higher electric resistivity. On the other hand, the nonequilibrium quasiparticle distribution close to the energy gap where the density of states is maximal can cause stimulation of superconductivity^13^. One can anticipate an interplay between the above mechanisms in a mixed state of *mw* driven type-II superconductors. However, we are not aware of a theory quantitatively interpreting our experimental results.

**Figure 4:**
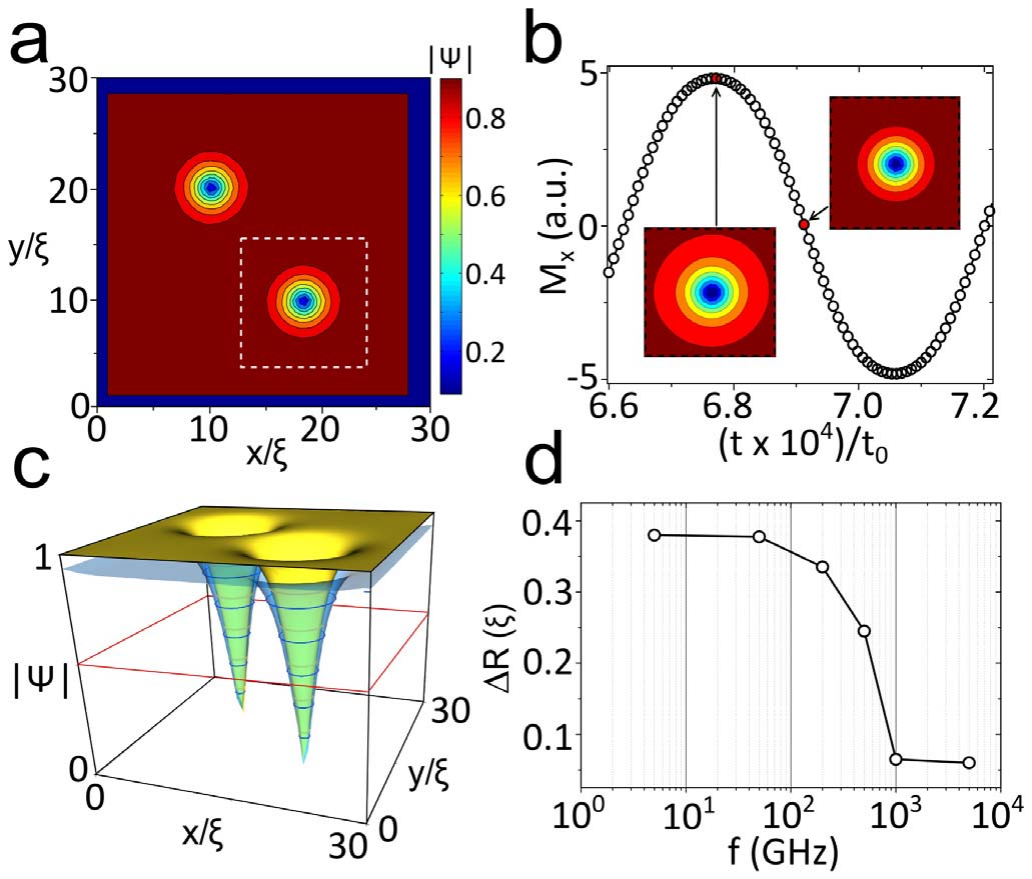
Time dependent Ginzburg-Landau simulations. (a) Contour plot of |Ψ| in a square sample, with *H_DC_* = 0.02*H_c_*_2_ perpendicular to the sample, after applying *H_DC_* = 0.8*H_c_*_2_ and slowly reducing it. Two vortices are isolated.(b) Oscillation of *M_x_* (component of magnetization in the direction of the ac field, in arbitrary units) as a function of time, following the ac field at 5 GHz. Snapshots of the area marked by dashed white lines in panel (a) show the different size of vortices at times separated by 1/4 of a period. (c) |Ψ| at the whole sample for the two cases considered in (b). Yellow surface corresponds to minimum and blue to maximum of |*H_DC_*|. (d) Change of vortex radius (taken at |Ψ| = 0.5, marked by red line in panel (c) as a function of frequency in an oscillation period. At high frequencies the vortices cannot follow the external field and their shape and position almost don't change.

To understand the nonlinear response of *mw* driven vortices we have simulated the *ac* response of vortices using the time dependent Ginzburg-Landau equations (see Suplementary material for details). The time derivative of the order parameter modulus |Ψ| shows that vortices oscillate about their equilibrium positions, especially at lower frequencies, when they are able to follow the external *ac* field without delay (Supplementary Fig. S9 b). As the *ac* field amplitude increases (which is equivalent to increasing the *mw* power of our measurements) the order parameter oscillates throughout the entire sample, being weaker at the maxima of amplitude of *mw* field *h_rf_*, as expected. This effect is specially pronounced in the outer part of vortex cores, as can be seen in Fig. 4b, c. When one compares the radius of a vortex (at a given value of, for example, |Ψ| = 0.5, see Fig. 4c) for different moments of an oscillation period, vortices are narrower at zero *ac* field amplitude than at its maximum *ac*. As happens in vortex cores displacement, the higher the frequency of the *ac* field, the more difficult is for a vortex core radius to change size (see Fig. 4d). A transition from linear to nonlinear response regimes is observed, *leading to a substantial reduction of the average vortex size* at high *mw* drives as a function of *f*. The reason for the different ranges of frequencies considered in the experiment and simulation are commented in the methods section.

The oscillations of the vortex core size under *mw* radiation are always present, and more notorious for higher *ac* field amplitudes, which is in qualitative agreement with the DC model by LO^30^. The above confirmation of LO-type mechanism in *ac* conditions agrees with simulations of DC driven vortices^34^ and does not exclude electron overheating in the vortex core as an additional factor contributing anomalous velocity dependence of vortex viscosity^29^.

The vortex velocity can be limited by the critical value *v_c_* for the LO instability^30^,^31^,^35^. Assuming that the maximum *mw*-induced shift of a vortex is limited by the inter-dot distance 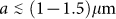 and that the dependence of *P*_O_ on *f* starts to saturate at 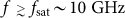 we estimate the geometrically restricted maximal vortex velocity as *v*_max_ = *f*_sat_ **·** *a* ≈ 6–10 km/s. This is 2–3 times larger than the values of *v_c_* reported for Nb and high-*T_c_* superconducting films^25^,^36^.

### Summary and conclusions

The experimental observation of stimulated superconductivity in type II superconductors has been used to quantify relative changes in vortex dissipation as a function of mobility (pinning). At high enough *mw* power and/or low enough *mw* frequencies (Fig. 3c) when MSSC is not effective, the vortex matching effects are clearly observed as periodic *dips in the mw losses* when *H*_⊥_ = *n*Φ_0_/*a*^2^ (*n* is an integer number and Φ_0_ the magnetic flux quantum). In contrast to that, in a broad range of *mw* powers (sufficiently below limiting values which heat the sample) and at high enough frequencies (above about 0.6 GHz) mobile (off-matching) vortices dissipate less than pinned vortices. One clearly observes *peaks in the vortex dissipation* in matching conditions. The higher the frequency, the broader the *mw* power range where matching anomalies are seen as peaks in losses. Microwave stimulation changes from dips at matching fields at the lowest frequencies to peaks at frequencies exceeding a few GHz, in agreement with TDGL simulations, that indicate a transition to a nonlinear regime when mobile (interstitial) vortices dissipate less than pinned ones. The observed effects (transition from peaks to dips) remain qualitatively unchanged for the range up to 3 vortices per pinning center, meaning that intervortex interaction has a weak influence on our resuts. The supplementary video 1 shows a simulation of the vortex response to a *mw* magnetic field. One clearly observes the changes of the vortex core radius and (through differential analysis of the modulus of the order parameter) the vortices motion. We find vortex deformation to be minimum because its displacement at *mw* frequencies is small in comparison with radius oscillations.

We point out that stray fields of Py dots do not play an essential role for the effects we observe. Unlike previous simulations^37^, our dots are in the magnetic vortex state with minimum stray fields^38^ and with ferromagnetic resonance (FMR) suppressed^39^, i.e., the dots are not saturated. Besides, there should be a strong structural pinning profile due to the fact that the SC film covers the array of dots and not vice versa as in Refs. 37. A qualitative similarity in the microwave losses measured with perpendicular or with inclined nearly parallel magnetic fields (Supplementary Fig. 8) further confirms that the observed effects are not induced by the presence of an in-plain component of magnetic field.

In conclusion, we have observed experimental signatures of stimulated superconductivity in type II superconductors in the vortex state including an enhancement of the upper critical fields and a somewhat less noticable increase of the critical temperature. Moreover, we have found experimentally and supported by simulations the unique fingerprint of MSSC in vortex dynamics -the reduced dissipation of microwave driven vortices due to a reduction of the vortex core size. Besides significance for condensed matter physics, our results may have implications for the current controversy on the possibility of stimulated superconductivity in holographic superconductors^20^,^21^.

## Methods

We investigated two types of samples: plain 60 nm thick Pb films 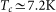 and 60 nm thick Pb films deposited over a square array of periodic pinning centers (Pb-PPC), consisting of circular Py dots (see Suplementary material for further details). All figures (except Fig. 2d and Supplementary Fig. S5) refer to the Pb-PPC sample.

The broadband measurements were done with a Vector Network Analyzer (VNA) connected to a coplanar waveguide (CPW) situated inside a cryostat with a superconducting magnet (see Suplementary material for details). The VNA signal excites the sample, placed on the CPW (Fig. 1a). The complex *mw* permeability, *U* ≡ *U*′ + *iU*″, is determined as the VNA transmission parameter *S*_21_, dependent on microwave power (*P*), frequency (*f*), temperature (*T*) and magnetic field (*H*), normalized by *S*_21_ at a reference *H* or *T*, in the normal state (Suplementary material for more details). Experimental figures corresponds to the estimated values of power waves travelling through the waveguide, but not absorbed by the vortex system.

To understand better the individual behavior of superconducting vortices under the influence of an in plane *ac* magnetic field, we have simulated the TDGL equation in 3D. Simulations allow to include a DC field perpendicular to the film to create vortices, and a sinusoidal field parallel to the plane that represents the *mw* field generated by the CPW. Both field components are introduced through the appropriate boundary conditions (see Suplementary material). Our simulations are based on the finite difference approach used several times in the past in 2D (see for example^32^). The Ginzburg-Landau parameter used is *κ* = 2. The temperature has been fixed far from *T_c_* = 7.2 *K* (*T* = 4 *K*) because vortices are better observed, being the results obtained still valid (although less vissible) at higher temperatures. A mismatch between the frequency range presented in the measurements and that of the simulations is due to the absence of precise knowledge of the characteristic time scales of the normal and superconducting parts of the GL simulation. We use our simulation just for qualitative confirmation of existence of LO mechanism for the microwave driven vortex. Future work could also try to analyze numerically possible coupled magnetic dot-superconducting vortex dynamics. This task, however, presents great challenge because of the need to include dynamics of magnetic pinning centers.

## Author Contributions

All authors discussed the results and commented on the manuscript. F.G.A. managed the project, A.L. performed microwave measurements and simulations, A.S. grew and characterized the samples, F.G.A. and A.L. wrote the manuscript with input from A.S. and V.V.M.

## Supplementary Material

Supplementary Information Supplementary Material

Supplementary Information Supplementary video 1

## References

[b1] Lindner N. H., Refael G. & Galitski V. Floquet topological insulator in semiconductor quantum wells. Nature Phys. 7, 490 (2011).

[b2] McIver J. W., Hsieh D., Steinberg H., Jarillo-Herrero P. & Gedik N. Control over topological insulator photocurrents with light polarization. Nature Nanotech. 7, 96 (2012). 10.1038/nnano.2011.214 22138862

[b3] Bergeret F. S., Virtanen P., Heikkila T. T. & Cuevas J. C. Theory of microwave-assisted supercurrent in quantum point contacts. Phys. Rev. Lett. 105, 117001 (2010). 20867598 10.1103/PhysRevLett.105.117001

[b4] Palomaki T. A., Harlow J. W., Teufel J. D., Simmonds R. W. & Lehnert K. W. Coherent state transfer between itinerant microwave fields and a mechanical oscillator. Nature. 495, 210 (2013). 23486060 10.1038/nature11915

[b5] Bhadrachalam P. *et al.* Energy-filtered cold electron transport at room temperature. Nature Comms. 5, 4745 (2014). 10.1038/ncomms5745 PMC4175579 25204839

[b6] Martínez-Pérez M. J. & Giazotto F. A quantum diffractor for thermal flux. Nature Comms. 5, 3579 (2014). 10.1038/ncomms4579 24694676

[b7] Wyatt A. F. G., Dmitriev V. M., Moore W. S. & Sheard F. W. Microwave enhanced critical supercurrents in superconducting constricted Tin films. Phy. Rev. Lett. 16, 1166 (1966).

[b8] Pals J. A. & Dobben J. Measurements of microwave-enhanced superconductivity in aluminum strips. Phys. Rev. B 20, 935 (1979).

[b9] Tolpygo S. K. & Tulin V. A. Influence of microwave irradiation on high-frequency absorption by thin superconducting films (superconductivity stimulation). Sov. Phys. JETP. 57, 123 (1983).

[b10] Heslinga D. R. & Klapwijk T. M. Enhancement of superconductivity far above the critical temperature in double-barrier tunnel junctions. Phys. Rev. B 47, 5157 (1993). 10.1103/physrevb.47.5157 10006681

[b11] Pals J. A. & Dobben J. Observation or Order-Parameter Enhancement by Microwave Irradiation in a Superconducting Aluminum Cylinder. Phys. Rev. Lett. 44, 1143 (1980).

[b12] Entin-Wohlman O. Comment on the observation of order-parameter enhancement by a change in the magnetic flux. Phys. Rev. B 23, 2428 (1981).

[b13] Eliashberg G. M. Film superconductivity stimulated by a high frequency field. JETP Lett. 11, 114 (1970).

[b14] Beck M. *et al.* Transient increase of the energy gap of superconducting NbN thin films excited by resonant narrow-band terahertz pulses. Phys. Rev. Lett. 110, 267003 (2013). 23848912 10.1103/PhysRevLett.110.267003

[b15] de Visser P. J. *et al.* Evidence of a nonequilibrium distribution of quasiparticles in the microwave response of a superconducting aluminum resonator. Phys. Rev. Lett. 112, 047004 (2014). 24580483 10.1103/PhysRevLett.112.047004

[b16] Abrikosov A. A. The magnetic properties of superconducting alloys. J. Phys. Chem. Solids 2, 199 (1957).

[b17] Hartnoll A., Herzog C. P. & Horowitz G. T. Building a Holographic Superconductor. Phys. Rev. Lett. 101, 031601 (2008). 18764244 10.1103/PhysRevLett.101.031601

[b18] Montull M., Pomarol A. & Silva P. J. Holographic Superconductor Vortices. Phys. Rev. Lett. 103, 091601 (2009). 19792782 10.1103/PhysRevLett.103.091601

[b19] Maeda K. & Okamura T. Vortex flow for a holographic superconductor. Phys. Rev. D 83, 066004 (2011).

[b20] Bao N., Dong X., Silverstein E. & Torroba G. Stimulated superconductivity at strong coupling. JHEP 10, 123 (2011).

[b21] Natsuume M. & Okamura T. The enhanced holographic superconductor: is it possible? JHEP 08, 139 (2013).

[b22] Gittleman J. I. & Rosenblum B. Radio-frequency resistance in the mixed state for subcritical currents. Phys. Rev. Lett. 16, 734 (1966).

[b23] Marcon R., Fastampa R., Giura M. & Silva E. Vortex-motion dissipation in high-*T_c_* superconductors at microwave frequencies. Phys. Rev. B 43, 2940 (1991). 10.1103/physrevb.43.2940 9997595

[b24] Golosovsky M., Tsindlekht M. & Davidov D. High-frequency vortex dynamics in *Y Ba*_2_*Cu*_3_*O*_7_. Supercond. Sci. Technol. 9 (1996).

[b25] Wördenweber R., Hollmann E., Schubert J., Kutzner R. & Panaitov G. Flux transport in nanostructured high-*T_c_* films at microwave frequencies. Physica C 479, 69 (2012).

[b26] Blatter G., Feigel'man M. V., Geshkenbein V. B., Larkin A. I. & Vinokur V. M. Vortices in high-temperature superconductors. Rev. Mod. Phys. 66, 1125 (1994).

[b27] Clem J. R. Local temperature-gradient contribution to flux-flow viscosity in superconductors. Phys. Rev. Lett. 20, 735 (1968).

[b28] Shekhter A., Bulaevskii L. N. & Batista C. D. Vortex viscosity in magnetic superconductors due to radiation of spin waves. Phys. Rev. Lett. 106, 037001 (2011). 21405287 10.1103/PhysRevLett.106.037001

[b29] Gurevich A. & Ciovati G. Dynamics of vortex penetration, jumpwise instabilities, and nonlinear surface resistance of type-II superconductors in strong rf fields. Phys. Rev. B 77, 104501 (2008).

[b30] Larkin A. I. & Ovchinnikov Y. N. Nonlinear conductivity of superconductors in the mixed state. Sov. Phys. JETP. 41, 960 (1976).

[b31] Doettinger S. G. *et al.* Electronic instability at high flux-flow velocities in high-*T_c_*, superconducting films. Phys. Rev. Lett. 73, 1691 (1994). 10056859 10.1103/PhysRevLett.73.1691

[b32] Buscaglia G., Bolech C. & López A. On the numerical solution of the time-dependent Ginzburg-Landau equations in multiply connected domains. Connectivity & Superconductivity, Berger, J. & Rubinstein, J. (Eds.), Springer (2000).

[b33] Coffey M. W. & Clem J. R. Theory of rf magnetic permeability of isotropic type-II superconductors in a parallel field. Phys. Rev. B 45, 9872 (1992). 10.1103/physrevb.45.9872 10000877

[b34] Vodolazov D. Y. & Peeters F. M. Rearrangement of the vortex lattice due to instabilities of vortex flow. Phys. Rev. B 76, 014521 (2007).

[b35] Silhanek A. V. *et al.* Influence of artificial pinning on vortex lattice instability in superconducting films. New Journal of Phys. 14, 053006 (2012).

[b36] Grimaldi G. *et al.* Magnetic field and temperature dependence of the critical vortex velocity in type-II superconducting films. J. Phys.: Condens. Matter 21, 254207 (2009). 21828431 10.1088/0953-8984/21/25/254207

[b37] Milosevic M. V. & Peeters F. M. Commensurate vortex configurations in thin superconducting films nanostructured by square lattice of magnetic dots. Physica C. 404, 246 (2004).

[b38] Gomez A. *et al.* Control of dissipation in superconducting films by magnetic stray fields. Appl. Phys. Lett. 102, 052601 (2013).

[b39] Aliev F. G. *et al.* Spin waves in circular soft magnetic dots at the crossover between vortex and single domain state. . Phys. Rev. B. 79, 174433 (2009).

