# Supplementary material for: Microwave-stimulated superconductivity due to presence of vortices
Source: Sci Rep. 2015 Mar 17;5:9187. doi: 10.1038/srep09187 (PMC4361862; doi:10.1038/srep09187)
Supplement: Supplementary Information — Supplementary Material [file srep09187-s1.pdf]

Microwave-stimulated superconductivity due to presence of vortices

A. Lara, F. G. Aliev
Dpto. Física Materia Condensada, Instituto Nicols Cabrera (INC) and Institute of Condensed Matter Physics (IFIMAC), Universidad Autónoma de Madrid, 28049, Madrid, Spain

Alejandro V. Silhanek
INPAC- Katholieke Universiteit Leuven, Celestijnenlaan 200D, B3001, Leuven, Belgium and Département de Physique, Université de Liège, B-4000 Sart Tilman, Belgium

Victor V. Moshchalkov
INPAC- Katholieke Universiteit Leuven, Celestijnenlaan 200D, B3001, Leuven, Belgium
(Dated: March 9, 2015)

I. SUPPLEMENTARY MATERIAL

Experimental details: The 60 nm thick Pb films were electron-beam evaporated onto liquid nitrogen-cooled Si/SiO_2 substrates. All samples were covered with a 20 nm thick protective layer of amorphous Ge, to avoid oxidation and keep them from getting scratched, since they are placed face down over the CPW. The Pb film is evaporated in all samples over a surface of 5×5 mm^2. In the Pb-PPC samples, the Pb films were deposited over a 2×2 mm^2 square array of 30 nm thick 1000 nm diameter Py dots, with an interdot distance of 2000 nm.

Hysteresis cycles confirmed that the Pb films are type II SC, in accordance with previous reports [1]. To reach the superconducting state, a JANIS helium cryostat with a superconducting magnet inside is used. Using two temperature control loops, the temperature stability is better than 0.2 mK and could be maintained up to 3 days.
The sample is placed at the end of an insert which contains a coplanar waveguide (CPW) to provide the microwave drive field ($\mathbf{h}_{\rm rf}$) up to about 0.1 Oe parallel to the plane. Supplementary Fig. S1 shows a calculation of the x and y components of the magnetic field generated near the central conductor, at a vertical distance of 20 nm for $P = 5$ dBm. The expressions found in [2] have been used for these calculations.

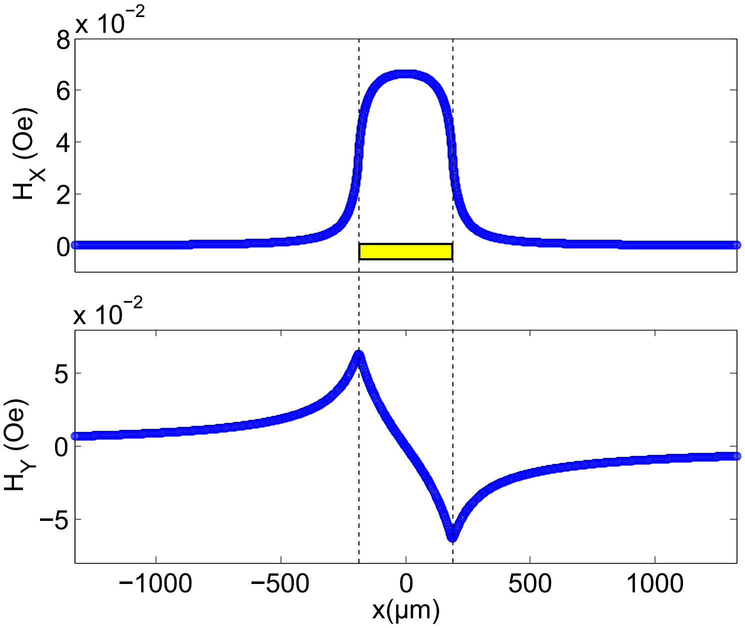

Suppl. Fig. 1. Magnetic field generated by the CPW at a vertical distance of 20 nm (sample surface) and $P = 5$ dBm. The yellow rectangle shows the dimensions of the central conductor of the CPW.

The CPW is designed to have a characteristic impedance of 50 Ω up to 25 GHz and emits mainly in the TEM mode. The central conductor is 375 μm wide, and the gap to ground planes is 140 μm. The CPW is made of Rogers laminates RO4350 10MIL/RO4003C 8MIL with a copper gladding (66 μm thick), and an extra gold plating on top. This waveguide does not excite the sample (placed on top of it) with a homogeneous field. Therefore, the vortices in different parts of the sample will feel a different amplitude and direction of the excitation. To account for how much vortices are excited on top of the central conductor, compared to far from it, in Suppl. Fig. 2 we show a histogram of the distribution of intensities of the microwave field. The largest value of field (corresponding to right in the middle of the central conductor) is found with a large frequency in the histogram, due to the almost parallel shape of the field close to this part of the waveguide. A large contribution can be seen in the histogram at low fields if long distances from the central conductor are considered in the calculations. With the appropriate weighting of each field with its frequency of appearance (inset of Fig. S. 2), we find that at a field H_x=0.034 Oe (corresponding to the edge of the CPW), this weighted signal equals 17% of the maximum, which is achieved at the center of the CPW. Therefore, vortices lying on top of the central conductor will be excited by an 83% of the total field delivered by the waveguide. The rest of the excitation lies beyond the central conductor.

More details on this setup can be found in [3]. The high frequency signal is emitted from one port of the Vector Network Analyzer, VNA, (an Agilent E8363C PNA model was used, with frequency range up to 40 GHz and power up to 7 dBm) and propagates through the CPW, to finally reach the other port.

The VNA emits a signal of some known power, but due to reflections inside the high frequency cables (two 1.5 m long cables in the insert to carry the signal inside the cryostat, and two 0.6 m long cables to connect them to the

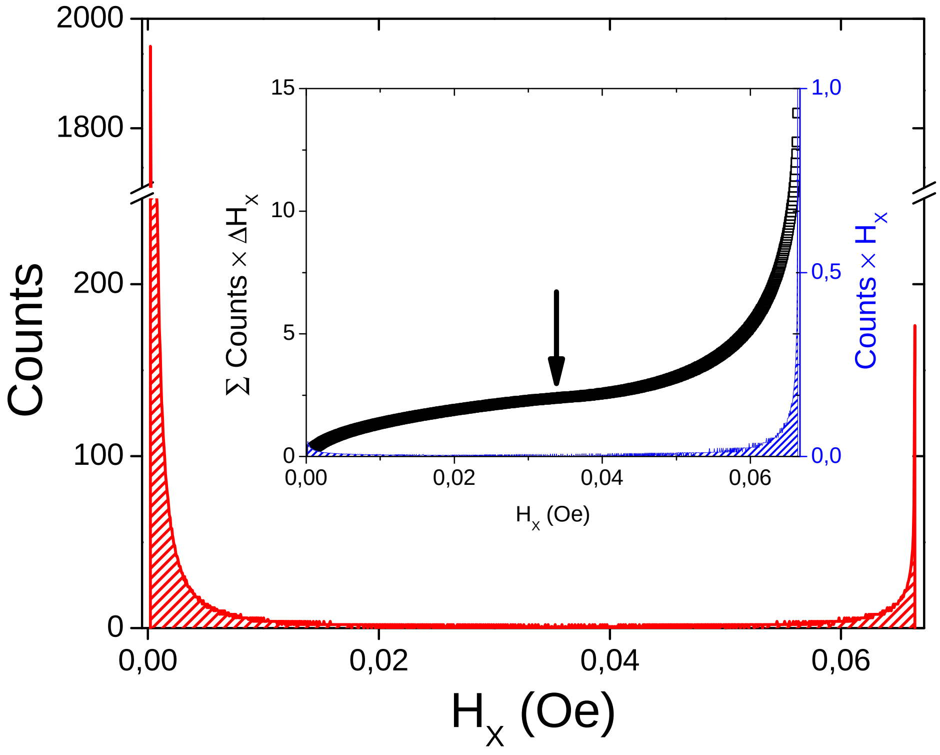

Suppl. Fig. 2. Histogram of distribution of magnetic microwave field created by the CPW. The inset shows a weighted distribution of fields (blue area) and a cumulative distribution. The arrow marks the field at the edge of the central conductor.

VNA) and connectors, and their heating due to eddy currents, microwave power is not fully transmitted from one port of the VNA to the other. The value of the signal frequency (f) is related to the appearance of internal reflections, and a f sweep gives the information of how much power is transmitted at every frequency by means of the $S_{21}(f)$ parameter. The power of the signal at the CPW, that we refer to as P in the article, is estimated as the average of the power emitted from port 1 and the power received at port 2, since the cables configuration connected to both ports is symmetric. We start from the definition of the transmission parameter in terms of voltages, when no signal comes out from port 2:

$$|S_{21}| = \left|\frac{V_{2,in}}{V_{1,out}}\right| \tag{1}$$

In this expression, $V_{2,in}$ refers to the voltage (amplitude and phase) of waves entering port 2, and $V_{1,out}$ to voltage of waves emitted from port 1. In terms of power, the expression for the amplitude is:

$$|S_{21}| = \sqrt{\frac{P_{2,in}}{P_{1,out}}} \tag{2}$$

Knowing $S_{21}(f)$, directly measured with the VNA, the power at port 2 is calculated as:

$$P_{2,in} = |S_{21}|^2 P_{1,out} \tag{3}$$

and the estimated power at the waveguide, P, is taken as the average:

$$P = \frac{P_{1,out} + P_{2,in}}{2} = P_{1,out}\frac{(1 + |S_{21}|^2)}{2} \tag{4}$$

It is to be noted that this P does not refer to the radiated electromagnetic energy incident on the sample, just to the power associated to the current that produces this radiation, as it propagates through the CPW.

This correction helps to quantify the losses due to the relation between wavelength and length of the cables. Higher frequency signals suffer more reflections, and are not so well transmitted to the second port, which can be quantified via S_{21}. However, more factors can contribute to decrease the power that is delivered to the sample, such as not perfectly symmetric cables, that would decrease the accuracy of the previous average. Also, it is important to remember that working at low temperatures can result in important differences compared to room temperature, due to changes in the length of cables, etc. In our case, this is not a problem, since the range of variation of T is small, always very close to T_c. Also, even if we could take into account all losses, the results presented in the main text are relative (throughout the range of powers considered, where transmission efficiency is invariant, since the frequency is fixed), and a correction of the absolute value of power will not affect the presence of the observed effects.

Measurement of the permeability parameter U: For the data analysis (i.e. calculation of the real and imaginary parts of U) the reflected signal is neglected (S_{11} coefficient of VNA), since its relative variation with respect to a reference trace is more than 15 times lower in magnitude than the transmitted signal (S_{21} coefficient). The same increase of T_c^* and H_{c2}^* at intermediate powers has been observed on the reflection coefficient S_{11} alone as well.
The permeability parameter was analyzed as the VNA transmission parameter (S_{21}) for every field normalized by the same parameter corresponding to the normal state of the sample, similar to the analysis method used in [4]:

$$\mu \propto U(f,P,H) = \frac{S_{21}(f,P,H)}{S_{21}(f,P,H_{\rm ref})}\,. \tag{5}$$

Here $S_{21}(f,P,H)$ and $S_{21}(f,P,H_{\rm ref})$ are the (f) and (P) dependent forward transmission parameters at the applied field of interest H, and the reference field $H_{\rm ref}$ (the maximum applied field, higher than H_{c2}). This expression is used when T is kept constant and H is changed. An analogous analysis is used in T sweeps at constant H, but normalizing at a reference temperature. It is necessary to achieve the normal state, either by increasing T or H, to have a clear signal corresponding to the SC response, when compared with the normal response, so well above T_c or H_{c2} conditions are necessary for a correct normalization. The quantity U is complex, and the imaginary part of $U = U' + iU''$, U'', represents microwave losses, while the real part U' represents energy stored and exchanged between sample and circuit (CPW), in this case flux screening by the superconductor.
Once U is found, values of T_c and H_{c2} can be found when reaching high enough values of T or H, so that the region in which the sample is in the normal state is clearly differentiated from the superconducting region. Since the transition is not perfectly sharp and well defined, the following method to determine T_c and H_{c2} will be used. To our best knowledge, there is not a universal criterion for extracting from permeability data the critical values of field and temperature. Therefore, in the following we will refer to the critical values obtained as T_c^* and H_{c2}^*, that may not be the exact values of critical T and H, but from which a clear dependence with microwave power is observed, that we interpret as stimulation of superconductivity by microwaves.
T_c^* is extracted from T sweeps at constant f and H, but varying P, so that MSSC can also be detected in these measurements. On the other hand, H_{c2}^* is found from H sweeps in which T and f are kept constant, while P also changes, to evidence the presence of MSSC not only from the values of T_c^*, but also from H_{c2}^*. For the results of T_c^* and H_{c2}^* discussed in this article, the real part of U was used, only because its changes are larger and easier to observe and analyze, but the superconducting transition is equally visible in U' and U''.

Critical temperature: The values of T_c^* were found in T sweeps by finding the intersection between piecewise polynomial fit of the response in temperatures for each power in both the superconducting part, and the normal part (see Suppl. Fig. 3 for an example of a T sweep at $f = 6$ GHz and $P = -17$ dBm, corresponding to Fig. 1c in the article). The resulting dependence of T_c^* on power clearly shows an increase at intermediate values of power. Analogous results (increase of T_c at intermediate powers) are also achieved when directly plotting the temperature at which the response changes in a (for example) 1% with respect to the normal state. The error in T_c^*, of 1 mK, is obtained statistically as the dispersion in the intersection, found by least squares.

Critical field: From measurements of $U(f,P,H)$ similar to those shown in Fig. 2a of the article (H sweeps) one can determine H_{c2}^* as the borderline separating the normal region from the superconducting one. As can be seen in Suppl. Fig. 4, showing a typical magnetic field dependence of $U'(f,P,H)$ at $P = -15$ dBm, $T = 7.17$ K and $f = 6$ GHz while changing the applied field, the transition from normal to superconducting state is not completely abrupt (the same happens when finding T_c^*). We define H_{c2}^* as the intersection of the polynomial low field fit of $U'(f,P,H)$ shown by a red solid line and the high field level corresponding to the normal state and fitted by blue lines.

The value of critical field for each microwave power is considered as the average of the values obtained for positive and negative fields, to cancel the shift produced by the magnetic field frozen in the superconducting magnet.
Analogous results (increase of H_{c2}^* at intermediate powers) are also achieved when directly plotting the field at which

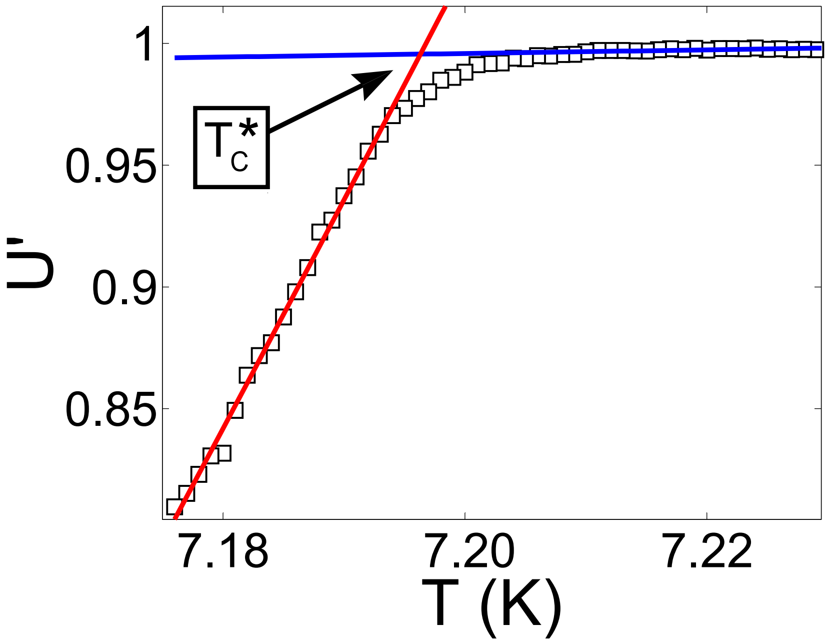

Suppl. Fig. 3. Method to determine T_c. In this case, U' is considered.

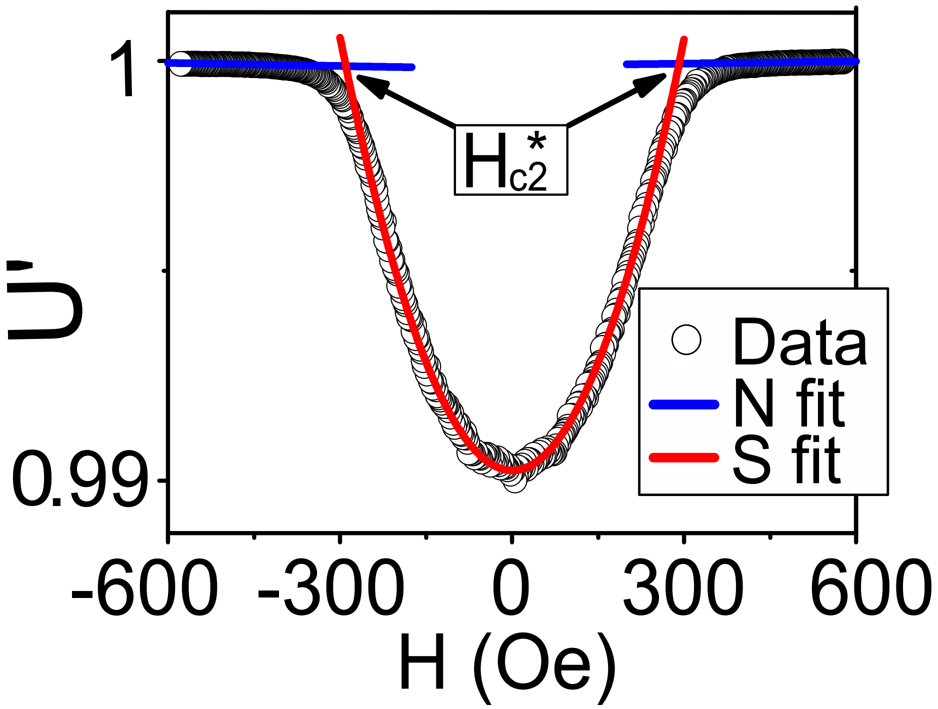

Suppl. Fig. 4. Method to determine H^*_{c2}

the response changes in a (for example) 0.01% with respect to the normal state.

Additional results and discussion:

Range of power considered: The VNA that we used can sweep power down to -40 dBm. We show results from -27 to 7 dBm. At powers lower than -27 dBm the sensitivity starts to decay rapidly, and there is noise that does not allow to have clear results. Also, a change of range is at -27 dBm, and some discontinuities may appear in the response. Despite the noise, the tendency at lower powers (not shown) is to present a constant signal (no changes of P_O are

observed in that region).

Influence of pinning: The article deals mainly with the sample with PPCs, since the stimulation of superconductivity appears to be stronger than in plain films. As a comparison, the same analysis presented in Fig. 2c in the article is shown in Suppl. Fig. 5 for the plain Pb film. It can be noted that the maximum relative upper critical field is lower in the plain films in comparison with Pb-PPC films. The introduction of pinning in Pb-PPC therefore seems to enhance the MSSC-related increase of H_{c2}^{*}. The experimentally observed stronger (and in a wider temperature interval) effects in the films with artificial vortex pinning could be linked with pairbreaking effects [5] from the Py dots stray fields [6]

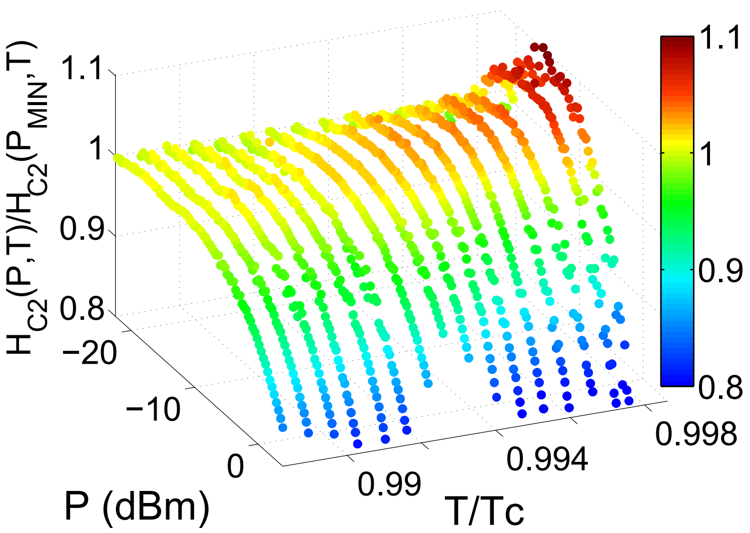

Suppl. Fig. 5. Relative critical field in the plain 60 nm thick Pb film

P_O close to H_{c2}^{*} far from T_c: Concerning the dependence of P_O on H, as explained in the article, it increases when increasing H. Close to T_c this trend is uniform, but at lower temperatures, and higher fields local maxima in optimum power appear (see Suppl. Fig. 6), as mentioned in the article, but not shown in Fig. 3b. We explain this behavior as a consequence of a dense vortex structure, when vortices are not able to move as freely as in cases with less density of them, diminishing the LO effect.

Anomalies in dissipation at matching fields: At frequencies below 2 GHz it is observed that matching anomalies transform from maxima to minima depending on f and P, at $T = 7.1$ K, as shown in Fig. 3 c) and Supplementary

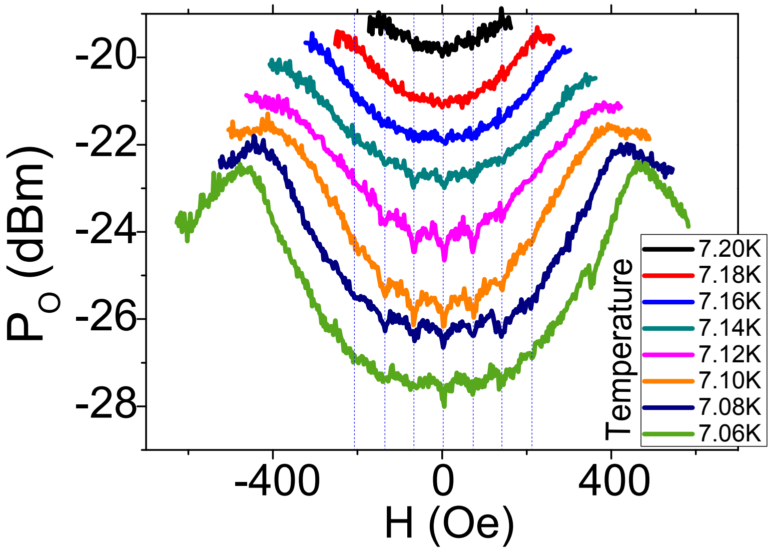

Suppl. Fig. 6. Optimum power as a function of magnetic field in the Pb-PCC film

Fig. S7 in the article.

In Figs. 3b and 3c in the main text and in Supplementary figures 6 and 7, matching fields have been indicated (*a postriori*) with doted lines. From these values we can verificate the interdot separation of the dots comprising the array of PPCs, as follows:

$$H_{\perp} = \frac{\phi_0}{a^2} \rightarrow a = \sqrt{\frac{\phi_0}{H_{\perp}}}$$

Our measurements in the case of field perpendicular to the film (Supplementary Fig. S8) give a value of matching fields of 4.8 Oe. With the standard value of the flux quantum, $\phi_0 = 2.06783 \cdot 10^{-15}$ Wb. Then, the interdot distance is, as expected:

$$a = \sqrt{\frac{\phi_0}{H_{\perp}}} = 2.075\mu m \simeq 2\mu m$$

Mainly the out of plane component creates the vortices that will give raise to matching conditions. Then, we can estimate the inclination of the field from the matching conditions. We know that matching fields for a perpendicular field occur at 4.8 Oe. Then, if we measure them at 72 Oe in the inclined configuration, as in Fig. 3b and 3c in the main text, we can extract the inclination angle as its sine, which equals the ratio of both quantities: $\sin\alpha = \frac{4.8}{72} = 0.066 \rightarrow \alpha = 3.78°$

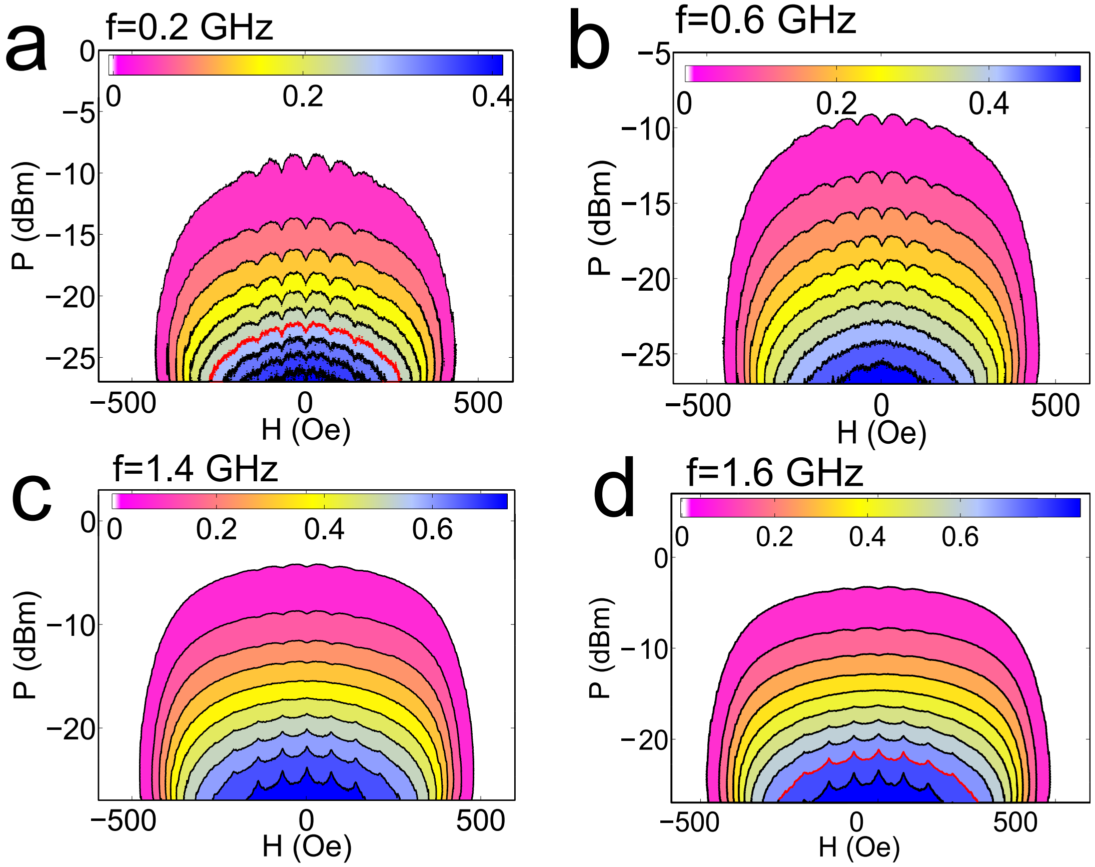

Suppl. Fig. 7. U'' as a function of H and P at different frequencies around 1 GHz. Panels a) and d) highlight a contour in red to stress the change from peaks to dips with frequency.

Dependence of the superconductivity stimulation on the magnetic field direction: Let us first discuss possible reasons why MSSC in the type II superconductors could be more clearly observed in an inclined magnetic field. We explain this observation as follows: As long as the applied microwave frequencies are substantially less than corresponding to

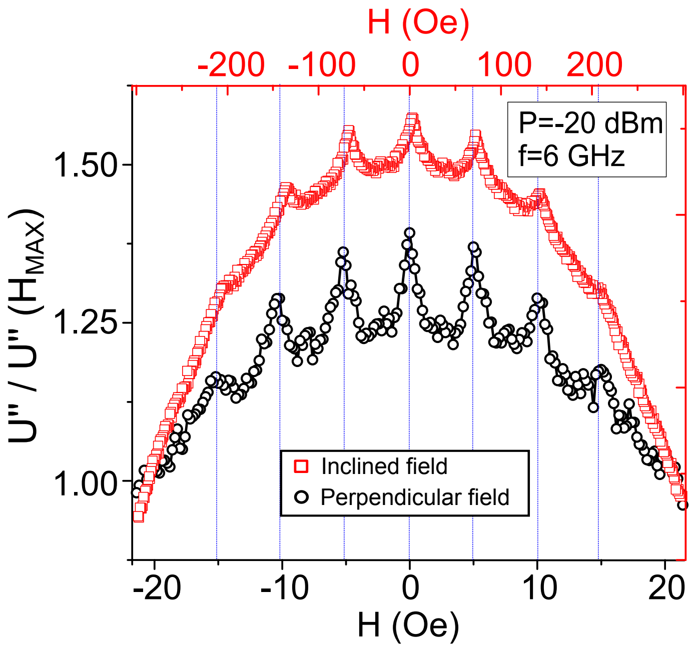

Suppl. Fig. 8. Comparison of matching fields in the case of perpendicular and inclined field.

the zero temperature gap values ($f_{Pb}(T = 0\text{K}) = 653$ GHz), and microwaves interact only with normal quasiparticles, the effectiveness of MSSC is conditioned by the presence of low energy normal quasiparticle states [7]. An additional (in-plane) external magnetic field effectively suppresses the order parameter, creating a gapless state in a rather extended field interval $\frac{H_{c2}}{2} < H < H_{c2}$ [5]. Then, for the suppressed order parameter values (i.e., close to T_c) the quasiparticles DOS varies roughly as $\sqrt{E}$ (here E is measured from the center of the gap) in the presence of impurities, providing effective quasiparticle excitation by subgap (GHz) microwaves [5]. Our experimental observations (Suppl. Fig. 6) reveal a clear enhancement of the MSSC strength in the form of an increase of P_{O} in the field interval ($\frac{H_{c2}}{3} < H < \frac{3}{5}H_{c2}$) followed by some small suppression of the P_{O} above $\frac{3}{5}H_{c2}$. Within our model, this increase is related in parts with an increase of the low energy quasiparticles DOS in the indicated field interval (mainly from in-plane field component) and enhanced contribution of the LO effects (from perpendicular field component) while vortex-vortex interaction remains unimportant.

It could be argued that the in-plane component of the inclined field can produce changes in the stray field of the dots, that would in turn affect the behavior of vortices. Experimental data of the dots shows that their annihilation field is higher than the values we work with in this case, so the stray field is always kept to a minimum. Direct measurements of U'' comparing the case of perpendicular and inclined field confirm that matching fields fall in the same values (obviously, after correspondingly scaling the fields, to match the perpendicular to the plane components, Supplementary Fig. 8)

Time dependent Ginzburg Landau simulations:

In order to understand the behavior of vortices under an in plane *ac* magnetic field, we have developed a computer program to simulate the 3D time dependent Ginzburg Landau (TDGL) equation. The equations, in the case of zero scalar potential gauge, can be expressed in a dimensionless form as follows [8]:

$$\frac{\partial \Psi}{\partial t} = -\frac{1}{\eta}\left[\left(-i\vec{\nabla} - \vec{A}\right)^2 \Psi + (1-T)\left(|\Psi|^2 - 1\right)\Psi\right] \tag{6}$$

$$\frac{\partial \vec{A}}{\partial t} = (1-T)\,\text{Re}\left\{\Psi^*\left(-i\vec{\nabla} - \vec{A}\right)\Psi\right\} - \kappa^2 \vec{\nabla}\times\vec{\nabla}\times\vec{A} \tag{7}$$

Where Ψ is the order parameter, $\vec{A}$ the vector potential and η a constant relating the relaxation times of normal and superconducting electrons. We use a finite difference scheme, a 2D version of which has been extensively used in

the past. However, the more complicated geometry we want to reproduce cannot be achieved in 2D, since magnetic flux (used for boundary conditions) needs at least two layers to be accomodated in the simulated domain (flux enters through a surface, therefore a single layer in 2D is not enough for considering in plane component of the magnetic flux, just perpendicular to the plane).
The equations integraged in time are four, one for the order parameter (first Ginzburg Landau equation), and the other three for the auxiliar variables known as link variables (see [8] for more details), that can be obtained by rearranging the three components of the second Ginzburg Landau equation for the vector potential. Link variables are defined as:

$$U^x_{x,y,z} = e^{-i\int_{x_0}^{x} A_x(\xi,y,z,t)d\xi}$$

$$U^y_{x,y,z} = e^{-i\int_{y_0}^{y} A_y(x,\eta,z,t)d\eta}$$

$$U^z_{x,y,z} = e^{-i\int_{z_0}^{z} A_z(x,y,\zeta,t)d\zeta}$$

Where x_0, y_0 and z_0 are arbitrary points of space that eventually cancel out. Given a plane, XY for example, the rectangle formed by the cells in (i, j), $(i+1, j)$, $(i, j+1)$ and $(i+1, j+1)$ will hold a circulation of the vector potential that can be related to magnetic flux at that position inside the superconductor:

$$\oint_{\partial\Sigma} \vec{A}\cdot d\vec{l} = \iint_{\Sigma} \vec{B}\cdot d\vec{s} = \Phi_B$$

From such a rectangle the discretized distribution of magnetic field can be found in the $x = i$, $y = j$, $z = k$ coordinates:

$$U^x_{i,j,k} U^y_{i+1,j,k} \overline{U^x}_{i,j+1,k} \overline{U^y}_{i,j,k} = e^{-iB(i,j,k)\Delta x \Delta y}$$

and a similar calculation for the other two planes YZ and ZX.

The geometry that we simulate is that of a thin film, with four cells in vertical direction. Doing so, magnetic flux is imposed according to boundary conditions (continuity of the parallel component of H) in the top and bottom planes, and allowed to evolve according to TDGL in the center of the film. The in plane dimensions are of $25\xi \times 25\xi \times 3\xi$, being ξ the coherence length, that is the unit of length of the simulations (its absolute value is not specified, it enters the computations through the Ginzburg Landau κ parameter). This is enough to distinguish clearly vortices from each other and from the borders, and at the same time small enough to allow for relatively fast computations. This is so because 3D version of TDGL needs to consider terms that 2D does not, since there are more types of possible border cells, making it inevitably slower to calculate than just N times slower, being N the number of cells in vertical direction. Details about the discretization of TDGL equations for a rectangular mesh can be found in [8].
By choosing an appropriate value of $\kappa = 2$, vortices appear when applying a perpendicular to the plane magnetic field above H_{c1}. This field is applied until the vortex lattice is stationary (vortices enter from the borders towards the center). Then, the *ac* magnetic field is applied parallel to the plane (keeping unchanged the perpendicular DC field). Keeping track of macroscopic quantities (such as the total magnetization) we can extract information about the global response of the system (vortices and the rest of the system, including borders), such as difference of phase with respect to external signal (for very high frequencies, vortices start to not being able to follow the field). However, we are more interested in the "microscopic" behavior of vortices, that can be studied by looking directly in the values of $|\Psi|$ at each simulation cell.
It has been found that vortices shape and position as a function of time depend both on frequency and amplitude of the external *ac* field. In general, under an in plane *ac* field vortices have been found to periodically shrink and widen in size (See Figure 4 in the main text). If microwave power (amplitude of the ac field) is large enough, these oscillations are seen very clearly. In Fig. 4d of the main text we show the variation in vortex core radius Δr at a fixed value of $|\Psi| = 0.5$ as a function of frequency for different powers. Higher powers allow to see better this difference, that is always present. The higher the difference of phase between *ac* field, and dynamic magnetization is, the more difficult is for the vortex to follow the external excitation, and these oscillations are not so well observed. As for the average redius of the vortex core, at higher frequencies it decreases, as predicted by LO, due to a redistribution of quasiparticles outside the vortex core.

It is to be noted that in solving TDGL equations, no energetic consdierations for stability of Cooper pairs is made, therefore effects such as Cooper pairs breaking due to absorption of photons of energy above the superconducting

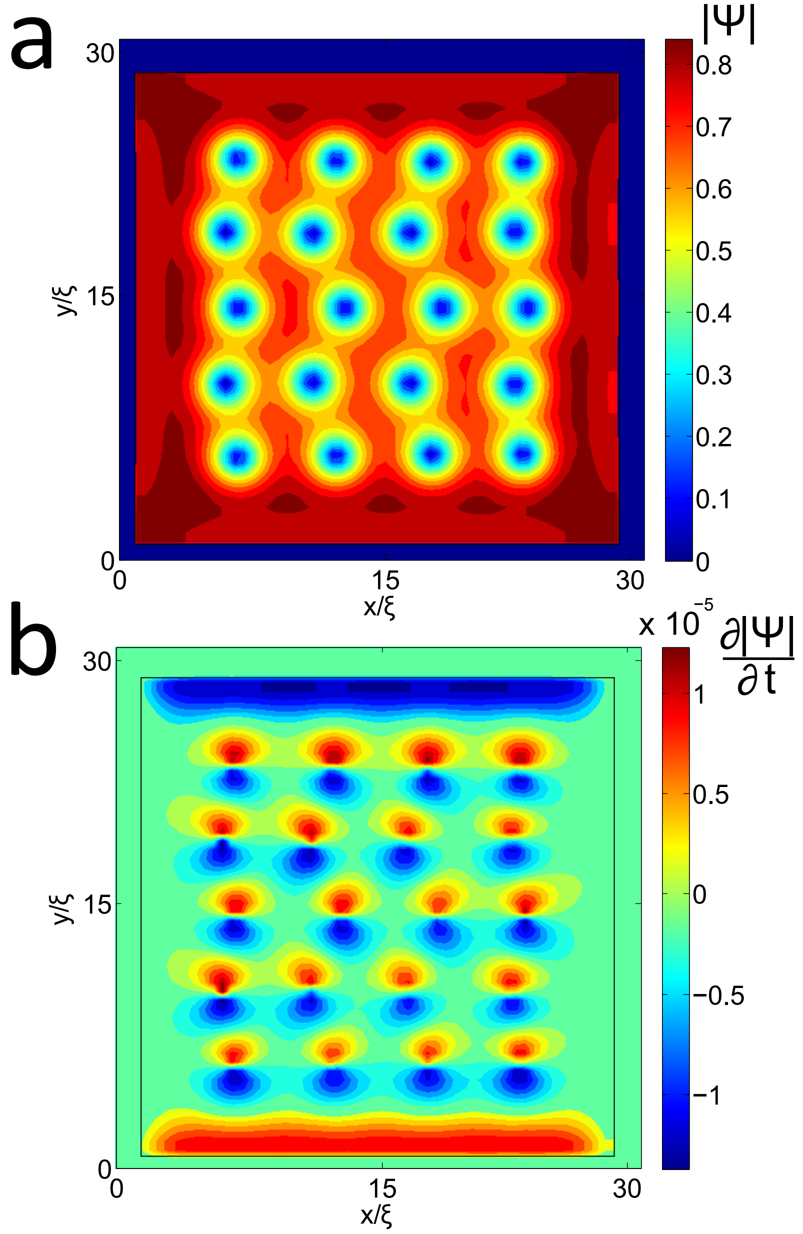

Suppl. Fig. 9. a) Magnitude of the order parameter for $H_{DC} = 0.4H_{c2}$. b) Time derivative of panel a). Vortices move toward the blue area. Half a period later, colors interchange

gap are not found. An arbitrarily high frequency *ac* field just makes it harder for vortices to follow it, not being frequency a cause of loss of superconductivity. On the other hand, a higher amplitude of the *ac* field does indeed destroy superconductivity. For example, at $|\mathbf{h}_{\rm rf}| = H_{c1}$, $|\Psi| < 1$ far from vortices. Thus, not only vortices are affected by large amplitude ac fields, also the rest of the film (as was already discussed before, concerning the excess of normal quasiparticles (unpaired electrons) when applying an inclined DC field).

It is important to note that in TDGL equations, there is no notion of Cooper pair, neither of their bonding energy. Typically, photons of energy in the THz range are energetic enough to break a Cooper pair when being absorbed by them, since this energy corresponds to the superconducting gap energy $\Delta \sim$meV. Therefore, even when we apply an *ac* field of frequencies the order of THz, the order parameter is not going to drop to zero because we are reaching the frequency corresponding to the superconducting gap. This concept is not considered in the equations we are solving. TDGL equations are useful for our purpose in that they give information on the redistribution of normal quasiparticles by applying an *ac* field to the vortex lattice, just by the deformation and movement of vortex cores. This redistribution of normal quasiparticles further interacts with the electromagnetic radiation of the waveguide, and

create extra losses due to Joule heating.

Influence of defects In the simulations, no periodic pinning centers are considered, we use them to study the reaction of vortices to a high frequency magnetic field.

However, the presence of defects can be considered. They are introduced by replacing the term $(|\Psi|^2 - 1)$ by $(|\Psi|^2 - r)$ (see [8]). Here, the term r is a variable that depends on position, and ranges between 1 and 0. By choosing values of r lower than 1, the superconductivity is weaker at those points, and vortices are in a more stable position, so it acts as a pinning center for vortices. In simulations we have used a defect of size ξ, with r=0 to trap a vortex. Several simulations at different frequencies show that regardless a vortex is inside a defect or not, the core size oscillates, as described in the main text. However, displacements of the vortex core are different, being more able to follow the external field when trapped. The vortices outside the defects also move but, especially at low frequencies, they tend to move in more circular trajectories, unlike vortices trapped in defects, which move more linearly. Also, radial oscillations of the vortex core size are observed both for vortices trapped inside defects, or outside defects, with a somewhat larger difference in amplitude at lower frequencies, as shown in Supplementary Fig. 10.

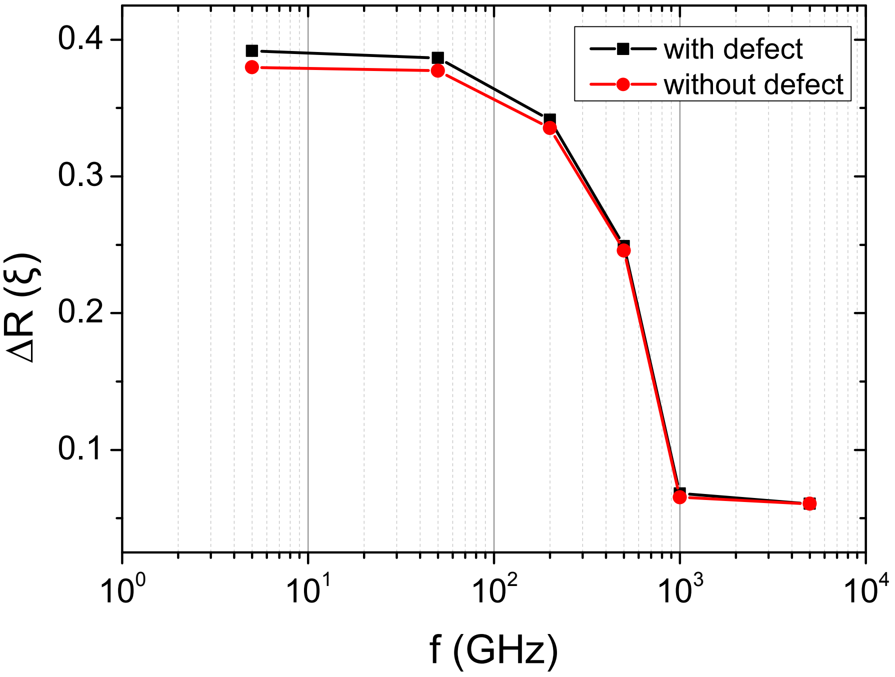

Suppl. Fig. 10. Frequency dependence of radial oscillations magnitude for a vortex trapped in a defect, or outside of it.

Description of supplementary video 1

The video shows the simulated response of two vortices to an external ac magnetic field. The left panel shows magnetization in the direction of the applied ac magnetic field as a function of time. The central panel shows contours of the order parameter. The right panel shows an image of the time derivative of the order parameter. The vortices motion is given by their instantaneous velocity. A vortex moves towards the brighter part of the time derivative of the order parameter, that alternatively changes according to the external field.

[1] Dolan, G. J. and Silcox, J. Critical Thicknesses in Superconducting Thin Films. *Phys. Rev. Lett.*, **30**, 603 (1973).
[2] Chumakov, D. et al. Nanosecond time-scale switching of permalloy thin film elements studied by wide-field time-resolved Kerr microscopy. *Phys. Rev. B* **71**, 014410 (2005)
[3] Awad, A. A. et al. Flux avalanches triggered by microwave depinning of magnetic vortices in Pb superconducting films. *Phys. Rev. B* **84**, 224511 (2011)

[4] Kuanr, B. K., Camley, R. E. and Celinski, Z. Extrinsic contribution to Gilbert damping in sputtered NiFe films by ferromagnetic resonance. *J. Magn. Magn. Mat* **286**, 276-281 (2005)

[5] Dukan, S. and Tesanovic, Z., Density of states of a type-II superconductor in a high magnetic field: Impurity effects. *Phys. Rev. B* **56**, 838 (1997).

[6] Lange, M., Van Bael, M. J., Bruynseraede, Y. and Moshchalkov, V. V. Nanoengineered magnetic-field-induced superconductivity. *Phys. Rev. Lett.*, **90**, 197006 (2003).

[7] Eliashberg, G. M. Film superconductivity stimulated by a high frequency field. *JETP Lett.* **11**, 114-116 (1970).

[8] Buscaglia, G., Bolech, C. and López, A. On the numerical solution of the time-dependent Ginzburg-Landau equations in multiply connected domains. *Connectivity & Superconductivity*, J. Berger and J. Rubinstein (Eds.), Springer (2000)
